# Differences in the clinical profile between isolated lupus anticoagulant and triplepositive antiphospholipid antibody profiles in primary antiphospholipid syndrome

**DOI:** 10.31744/einstein_journal/2026AO1654

**Published:** 2026-10-13

**Authors:** Bruno Verza Azevedo, Flavio Signorelli, Gustavo Guimarães Moreira Balbi, Danieli Castro Oliveira Andrade

**Affiliations:** 1 Universidade de São Paulo Faculdade de Medicina Hospital das Clínicas São Paulo SP Brazil Hospital das Clínicas, Faculdade de Medicina, Universidade de São Paulo, São Paulo, SP, Brazil.; 2 Universidade do Estado do Rio de Janeiro Hospital Pedro Ernesto Rio de Janeiro RJ Brazil Rheumatology Service, Hospital Pedro Ernesto, Universidade do Estado do Rio de Janeiro, Rio de Janeiro, RJ, Brazil.; 3 Universidade Federal de Juiz de Fora Hospital Universitário Juiz de Fora MG Brazil Rheumatology Service, Hospital Universitário, Universidade Federal de Juiz de Fora, Juiz de Fora, MG, Brazil.

**Keywords:** Antiphospholipid syndrome, Lupus coagulation inhibitor, Thrombosis, Beta 2-Glycoprotein I, Cardiolipins

## Abstract

**Objective:**

To compare the clinical phenotype of patients with primary antiphospholipid syndrome who have isolated lupus anticoagulant positivity *versus* those with triple positivity.

**Methods:**

This cross-sectional study included two cohorts from two tertiary centers in Brazil comprising patients diagnosed with primary antiphospholipid syndrome.

**Results:**

Of 313 patients, 135 were selected and divided into two groups. A total of 99 had isolated lupus anticoagulant positivity and 36 had triple positivity. Most participants were female (58.5%) and White (61.5%), with a mean age of 50.1±12.0 years and a median disease duration of 16 (12-22) years. Both groups showed similar rates of thrombosis (93.9% in the lupus anticoagulant group *versus* 100% in the triple positivity group, p=0.131) and obstetric events (55.8% *versus* 60.0%, p=0.754). Microthrombotic and non-thrombotic event rates were also comparable. Hydroxychloroquine use was higher in the triple-positive group (36.1%) than in the lupus anticoagulant group (15.2%, p=0.008).

**Conclusion:**

No more severe phenotype was observed in patients with triple positivity compared with those with isolated lupus anticoagulant positivity. The mixed ancestry and demographic composition of the Brazilian population may explain differences compared with more homogeneous populations.

## Highlights

■ Similar rates of thrombosis and adverse pregnancy outcomes were observed in the lupus anticoagulant-only and triple-positive groups.
■ Hydroxychloroquine use was higher in the triple-positive group.
■ No statistically significant differences were observed in microvascular findings between the two groups.

## In Brief

In Brazilian cohorts, patients with isolated lupus anticoagulant and those with triple-positive profiles exhibited similar clinical outcomes, with no statistically significant differences in the prevalence of venous or arterial thrombosis or in rates of fetal loss and miscarriages. Although triple positivity is typically associated with a worse prognosis, the only notable difference observed was a higher frequency of hydroxychloroquine prescription in this group, likely due to perceived increased risk. These findings suggest that the severity attributed to triple positivity may be largely driven by the presence of lupus anticoagulant itself. Furthermore, the results differ from those reported in other populations, possibly due to the genetic diversity of the Brazilian population, highlighting the need for further comparative studies across antiphospholipid syndrome profiles.

## Graphical abstract

**Figure:**
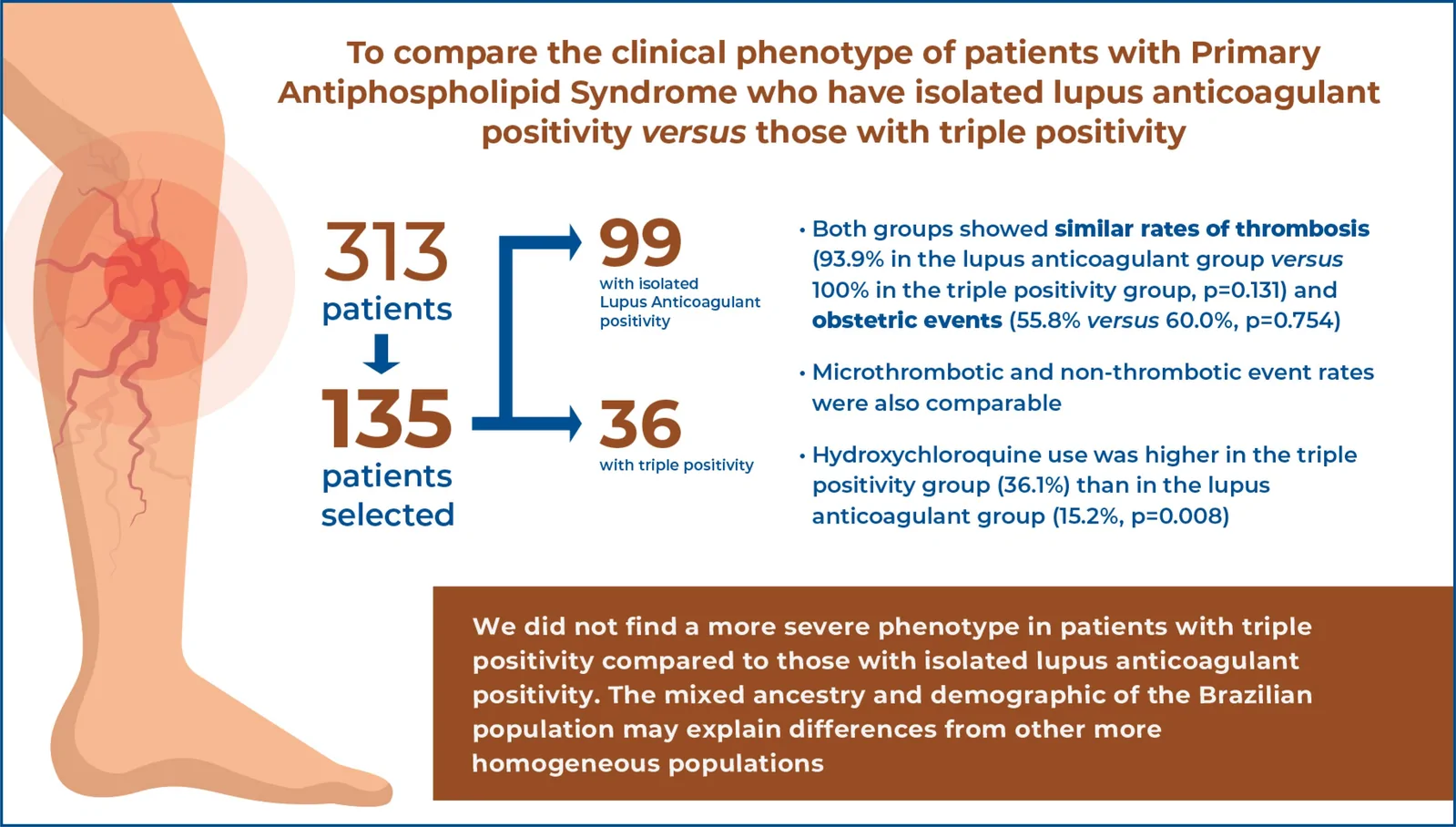

## INTRODUCTION

Antiphospholipid syndrome (APS) is an immune-mediated disease characterized by the occurrence of macro- and microvascular thrombotic events, non-thrombotic manifestations, and pregnancy morbidity, associated with the persistent presence of antiphospholipid antibodies (aPL), namely anticardiolipin (aCL), anti-β2-glycoprotein-1 (anti-β2GPI), and lupus anticoagulant (LA).^(1)^ According to its association with other autoimmune rheumatologic diseases (typically systemic lupus erythematosus [SLE]), it may be further classified as secondary APS (SAPS) or primary APS (PAPS) when isolated. Thrombotic manifestations can be arterial, venous, or microthrombotic, with deep vein thrombosis and stroke being the most common venous and arterial thrombotic events, respectively.^(2)^ The new classification criteria endorsed by the American College of Rheumatology and the European Alliance of Associations for Rheumatology (ACR/EULAR) defined microthrombotic manifestations, including livedo racemosa, livedoid vasculopathy, nephropathy, suprarenal hemorrhage, alveolar hemorrhage, and microvascular myocardial infarction.^(1)^ Major non-thrombotic manifestations include valvulopathy and thrombocytopenia, whereas some manifestations, such as Raynaud's phenomenon and neurological symptoms such as migraine, chorea, and myelitis, are not covered by the Sydney criteria or the new ACR/EULAR 2023 criteria.^(1,2)^

Many studies have indicated that patients with triple positivity (TP) for aPL are more prone to experience thrombotic events. Pengo et al. showed that patients with APS and TP had almost fourfold higher risk of cardiovascular events than those with other aPL profiles.^(3)^ In a further analysis, nearly half of the TP patients had a vascular event after 10 years of follow-up.^(4)^ In fact, when applying the adjusted Global AntiphosPholipid Syndrome Score (aGAPSS), a tool developed for thrombosis risk prediction in patients with APS, the presence of all three aPL criteria was assigned a higher score than the presence of each aPL criterion individually.^(5,6)^ More recently, Laurent et al. reported that TP patients presented with lower relapse-free survival rates than patients without TP in a French cohort.^(7)^

In contrast, another common description in the literature is the higher risk of thrombotic events in patients with isolated LA compared with groups positive for aPL identified by solid-phase assays. These individuals have a greater risk of thrombosis at any vascular site and worse survival rates. ^(8–10)^ In accordance with these findings, the new ACR/EULAR classification criteria for APS assigned a weight of five to persistent LA positivity, only comparable with high-positive IgG aCL or anti-β2GPI levels (≥80U).^(1)^

Recently, Galli et al. analyzed the clinical and laboratory differences between Brazilian and non-Brazilian patients included in the Anti-Phospholipid Syndrome Alliance for Clinical Trials and InternatiOnal Networking (APS ACTION) cohort. Brazilian patients were more likely to be single positive for LA (51.1% *versus* 19.6%, p<0.001) and less likely to be TP (8% *versus* 41.6%, p<0.001).^(8)^ However, no statistically significant differences in the clinical and obstetric manifestations were found between the groups.

Based on the aforementioned data, this study aimed to verify whether clinical differences exist between patients with PAPS who are positive for LA alone (isolated LA) *versus* those with TP.

## OBJECTIVE

To compare the clinical phenotype of patients with primary antiphospholipid syndrome who have isolated lupus anticoagulant positivity *versus* those with triple positivity.

## METHODS

This was a cross-sectional study conducted in two APS outpatient clinics (*Hospital Universitário Pedro Ernesto/Universidade Estadual do Rio de Janeiro* [UERJ] and *Hospital das Clínicas/Universidade de São Paulo* [USP]). Patients diagnosed with PAPS who attended the clinics between August 2016 and July 2023 were screened. For those with more than one visit, the last outpatient visit was considered the index visit (baseline); therefore, cumulative clinical and laboratory information was collected at that visit. The diagnosis of PAPS was considered irrespective of when it was established (previously or during data collection). Our present study was conducted between July 2022 and July 2023, and patients who fulfilled the Sydney classification criteria were consecutively selected.^(2)^ Patients with incomplete aPL profiles (one of the aPLs not performed) were excluded. However, patients without confirmation of one of the aPLs (positive for only one of them) were also excluded. Only patients with isolated positivity for LA (confirmed at least twice) and TP (all three aPLs confirmed at least twice) were included. Other aPL profiles confirmed at least twice, namely isolated positivity for aCL or anti-β2GPI, and double positivity (any positive combination of two aPLs), were not eligible. All procedures were performed in accordance with the Declaration of Helsinki and the ethical standards of the responsible committee on human experimentation. The study protocol was approved by the Institutional Ethics Committee of *Hospital das Clínicas*, *Faculdade de Medicina, Universidade de São Paul*o (CAAE: 59061422.5.0000.0068; #5.516.598). Patient data were analyzed anonymously, and no interventions were made; therefore, informed consent was not required. Data collection was performed similarly in each cohort using a structured database.

Patients were divided into two groups according to their aPL profile: isolated positivity for LA and TP. Lupus anticoagulant testing was conducted according to the International Society on Thrombosis and Hemostasis guidelines applicable at each evaluation time in both university hematology core laboratories.^(11)^ The assays performed at the USP laboratory for IgG and IgM aCL were conducted using Phadia EliA^®^ fluorescence enzyme immunoassays (Thermo Fisher Scientific). The IgG and IgM anti-β2GPI assays were conducted using QUANTA Lite® anti-β2GPI ELISA kits (INOVA Diagnostics). Regarding the assays performed at the UERJ lab, IgG and IgM aCL were determined using QUANTA-Lyser 240® (Werfen). The IgG and IgM anti-β2GPI assays were conducted using EUROLabWorkstation Elisa® (Euroimmun). The aCL isotypes were considered positive if they exceeded 40U/mL (or the 99^th^ percentile), and anti-β2GPI positivity was defined as values above the 99^th^ percentile (Sydney criteria).^(2)^ All aPLs were confirmed with a minimum of 12-week intervals. High levels of aCL and anti-β2GPI were defined as ≥80IU/mL, as recently suggested by the 2023 ACR/EULAR classification criteria.^(1)^

The two groups were compared with regard to demographic and clinical characteristics. Race was categorized as White and non-White based on self-declaration. We included venous and arterial thrombosis (confirmed by appropriate testing), pregnancy morbidity (three or more unexplained consecutive spontaneous abortions before the 10th week of gestation, with maternal anatomic or hormonal abnormalities and paternal and maternal chromosomal causes excluded or one or more unexplained deaths of a morphologically normal fetus at or beyond the 10th week of gestation or one or more premature births of a morphologically normal neonate before the 34th week of gestation because of eclampsia or severe pre*-*eclampsia defined according to standard definitions or placental insufficiency),^(2)^ presence of livedo racemosa (unexplained violaceous, "net-like," blotchy mottling of the skin with nonuniform, irreversible, broken, and asymmetric persistent discoloration), aPL-associated nephropathy (defined as one of the following unexplained persistent clinical or laboratory findings: new-onset hypertension or deterioration of previously well-controlled hypertension; proteinuria ≥0.5g in 24-hour urine specimen or protein:creatinine ratio ≥0.5mg/mg; acute renal failure; or glomerular microscopic hematuria), thrombocytopenia (an otherwise unexplained platelet count of <100×10^9^/L on at least two occasions), and valvulopathy (valve thickening or vegetation on echocardiogram).^(1)^ Cardiovascular risk factors for thrombosis were also evaluated: hypercholesterolemia, arterial hypertension, and diabetes. The definitions of these factors are described elsewhere according to current guidelines^(12–14)^. Other risk factors included obesity (defined as body mass index ≥30kg/m^2^) and smoking (ever). Antiphospholipid syndrome treatment at the time of the study, including low-dose aspirin, warfarin, heparin, and hydroxychloroquine, was also recorded.

Statistical analyses were performed using SPSS software (version 23; Chicago, IL, USA). Bivariate analyses were conducted using Fisher's exact test or χ^2^ test for categorical variables and the Mann-Whitney U test or Student *t*-test for continuous variables. Normality was evaluated using the Shapiro-Wilk test and graphical analyses. Results with a (p<0.05) were considered significant.

## RESULTS

### Patient selection

Initially, we screened 313 patients followed in our APS outpatient clinics. We excluded 178 patients. Five patients were excluded due to another autoimmune rheumatic disease (3 SLE and 2 Sjögren's disease). Five patients had aPL-related thrombocytopenia, one had aPL-related superficial venous thrombosis, and five were aPL-negative and were diagnosed with so-called seronegative APS. Subsequently, we excluded 23 patients with incomplete aPL profiles (six without LA, two without aCL, and 15 without anti-β2GPI). We excluded 104 patients without confirmatory positive results for any aPL. The remaining 35 excluded patients had aPL profiles other than triple positivity or isolated positivity for LA. Isolated positivity was present in one patient for anti-β2GPI and in four patients for aCL, respectively. Double positivity was distributed as follows: 14 for both LA and aCL, 13 for both LA and anti-β2GPI, and three for both aCL and anti-β2GPI. We included 135 patients (58.5% from UERJ and 41.5% from USP), of whom 36 were triple positive and 99 had isolated LA positivity. Most were female (58.5%), White (61.5%), with a mean age of 50.1±12.0 years and a median disease duration of 16 (12-22) years. A flowchart of the study is shown in figure 1.

**Figure 1:**
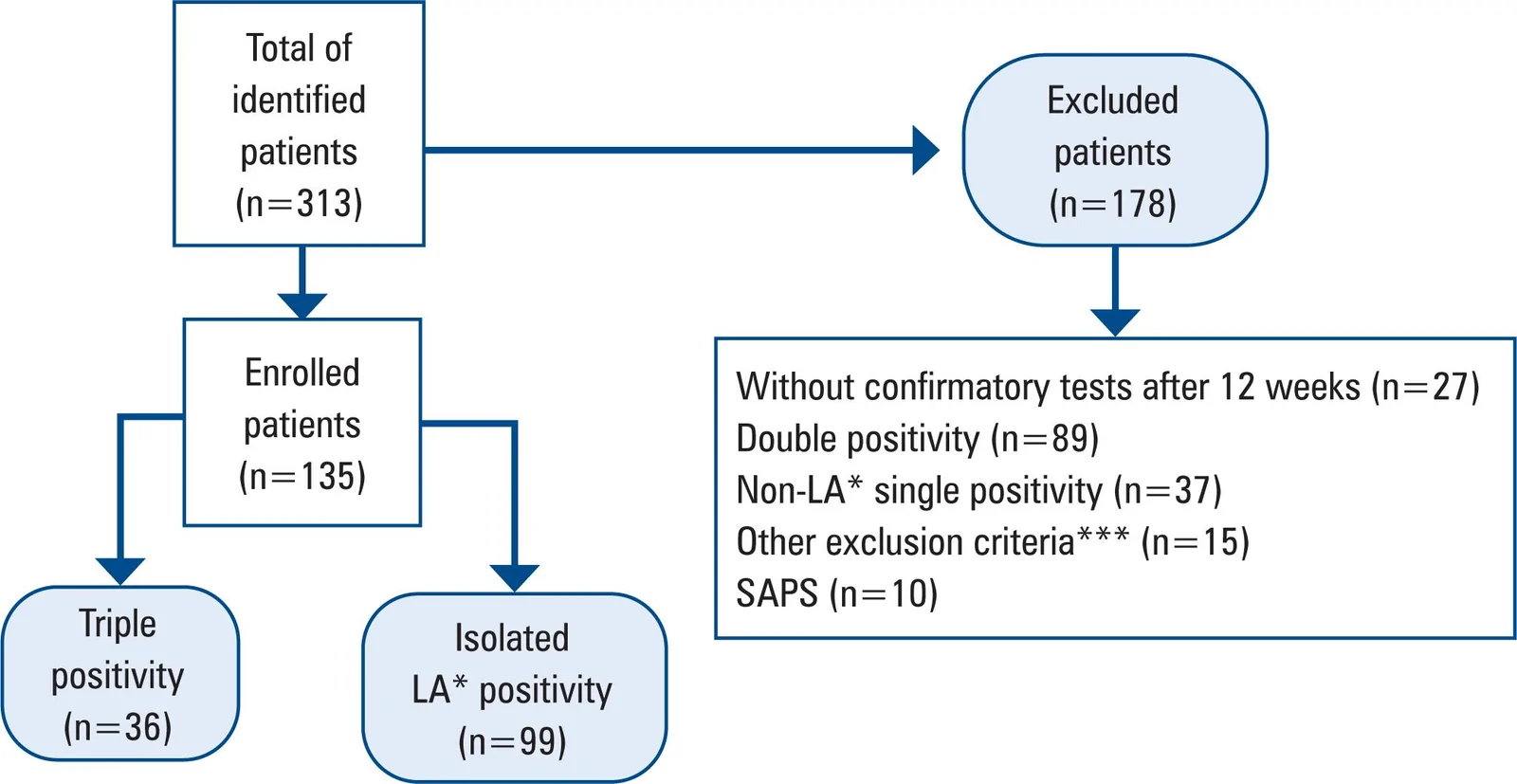
Flowchart of patients inclusion

* patients diagnosed by another method.

LA: lupus anticoagulant; SAPS: secondary antiphospholipid syndrome.

### Comparison between triple-positive and isolated lupus anticoagulant patients

Demographic characteristics showed that patients in the isolated LA Group were more likely to be female (63.6% *versus* 44.4%, p=0.045) and non-White (44.4% *versus* 22.2%, p=0.019) compared with the TP Group. The median age at inclusion and at APS diagnosis were similar between groups.

Antiphospholipid syndrome clinical features did not differ significantly between groups. The rates of thrombotic events were 93.9% in the isolated LA Group and 100% in the TP Group (p=0.131), while obstetric events were 55.8% and 60%, respectively (p=0.754). Rates of recurrent abortions (20% *versus* 7.1%, p=0.316), fetal loss (28.5% *versus* 14.1%, p=0.845), and prematurity (25.3% *versus* 12.6%, p=0.858) were also similar between Groups. Hydroxychloroquine use was less common in the isolated LA group than in the TP Group (15.2% *versus* 36.1%, p=0.008). All cardiovascular risk factors were similar between the two Groups. A comparative analysis of patients with isolated LA and TP is presented in table 1.

**Table 1:** Comparison of the main characteristics between isolated lupus anticoagulant positivity and triple positivity groups

		Isolated LA positivity (n=99)	Triple positivity (n=36)	p value
Demographic characteristics				
Age, median (IQR)		48 (41-60)	47 (40-56)	0.217
Time since diagnosis, median (IQR)		16.1 (12.1-22.6)	16.4 (12.1-21.1)	0.737
Gender (female), n (%)		63 (63.6)	16 (44.4)	0.045
White, n (%)		55 (55.6)	28 (77.8)	0.019
	Age at first manifestation, median (IQR)	31 (24-41)	28.5 (24-33.8)	0.362
Macrovascular manifestations - n (%)				
	Thrombotic events	93 (93.9)	36 (100)	0.131
	Arterial event	36 (36.4)	16 (44.4)	0.394
	Stroke	22 (22.2)	10 (27.8)	0.502
	Venous	73 (73.7)	25 (69.4)	0.621
Pregnancy morbidity				
	Gestational events, n (%)*	24 (55.8)	12 (60.0)	0.754
	Three consecutive abortions, n (%)**	3 (20.0)	1 (7.1)	0.316
	Fetal loss, n (%)*	18 (28.5)	9 (14.1)	0.845
	Prematurity, n (%)*	16 (25.3)	8 (12.6)	0.858
Microvascular manifestations - n (%)				
	Livedo	21 (21.2)	12 (33.3)	0.147
	Skin ulcer	6 (6.1)	0 (0)	0.342
	Nephropathy	5 (5.1)	2 (5.6)	0.657
Non-thrombotic manifestations - n (%)				
	Thrombocytopenia	6 (6.1)	6 (16.7)	0.083
	Valvulopathy***	13 (14.9)	5 (16.7)	0.855
Treatment - n (%)				
	Acetylsalicylic acid	26 (26.3)	5 (13.9)	0.131
	Warfarin	89 (89.9)	34 (94.4)	0.515
	Hydroxychloroquine	15 (15.2)	13 (36.1)	0.008
Traditional cardiovascular risk factors - n (%)				
	Hypertension	43 (43.4)	18 (50)	0.498
	Diabetes mellitus	11 (11.1)	1 (2.8)	0.181
	Hypercholesterolemia	47 (47.5)	13 (36.1)	0.240
	Smoking	37 (37.4)	10 (27.8)	0.301
	Obesity	48 (48.5)	16 (44.4)	0.678

* Only female patients were included in this analysis (n=63): 43 in the LA Group and 20 in the TP Group (male and nulliparous patients were excluded);

** Only patients with ≥3 pregnancies were included in this analysis (n=29): 15 in the LA Group and 14 in the TP Group;

*** Only patients evaluated by echocardiogram were included in this analysis (n=117): 87 in the LA Group and 30 in the TP Group.

LA: lupus anticoagulant; TP: triple positivity; IQR: Interquartile range.

### Titers of antiphospholipid antibodies measured by ELISA in triple-positivity patients

A noteworthy finding was that aPL titers measured by ELISA were frequently high in TP patients (Figure 2). Of the 36 patients in this group, 30 (83.3%) and 23 (63.9%) exhibited high levels of anti-β2GPI and aCL, respectively.

**Figure 2:**
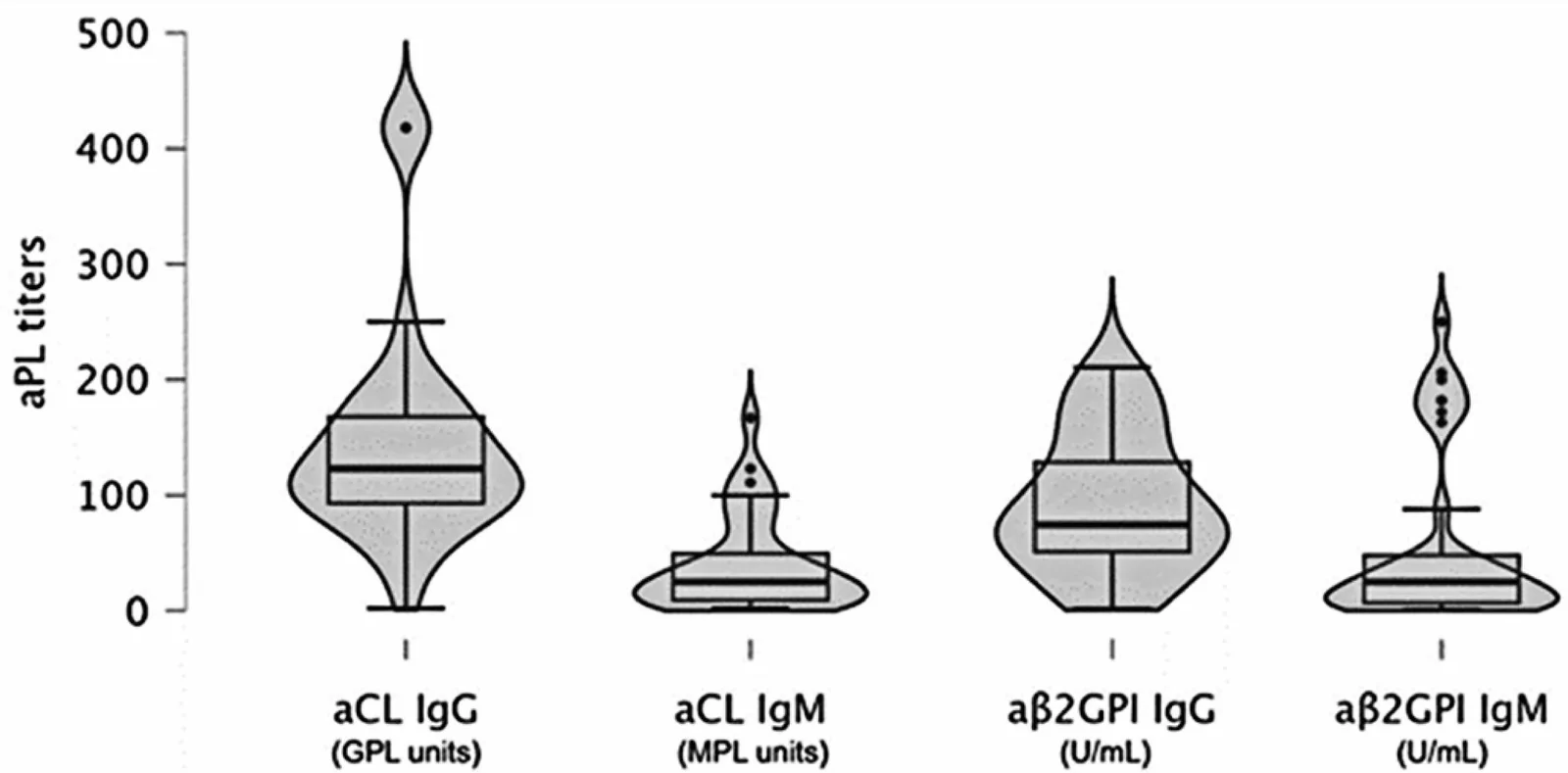
Antiphospholipid antibodies titers in triple-positive patients

aCL: anticardiolipin; aPL: antiphospholipid antibodies; anti-β2GPI: anti-beta-2 glycoprotein I.

This figure shows a combination of box and violin plots of solid-phase assay levels of antiphospholipid antibodies in patients with triple positivity. Dots represent outliers.

## DISCUSSION

Although there is a general notion that the risk of recurrent thrombosis is higher in patients with TP than in those with isolated LA, we could not demonstrate any differences between these two groups when thrombotic, microthrombotic, non-thrombotic, and obstetric events were compared.

Lupus anticoagulant positivity is a well-established risk factor for thrombosis in APS cohorts worldwide.^(8)^ In a meta-analysis, Galli et al. showed the importance of LA in thrombosis prediction when compared with aCL. Of 52 prospective, case-control, and cross-sectional studies published between 1998 and 2000, five analyzed the odds ratio (OR) of both LA and aCL for thrombosis. All five studies found a significant association between LA and thrombosis (OR ranging from 5.71 to 9.4), while none identified an association between aCL and thrombotic events.^(8)^ Despite the importance of anti-β2GPI to current understanding of the syndrome's pathogenesis,^(15)^ its association with arterial complications, such as myocardial infarction and acute stroke, was weaker than that of LA.^(16)^

Although TP has been associated with a higher risk of thrombosis recurrence than other aPL profiles in studies conducted mainly in Europe,^(3,4,5,7)^ patients in our cohort who were LA-positive had a similar clinical phenotype to those who were positive for TP. Based on this, we hypothesized that this lack of difference between TP and isolated LA may be due to genetic background and/or racial admixture. Our hypothesis was supported by a study conducted by Hernández-Molina et al., which analyzed 95 Mexican patients with PAPS and identified that the effect size for thrombosis recurrence was higher in LA-positive patients (OR=3.6, 95%CI=1.1-11.2, p=0.03) than in TP patients (OR=3.2, 95%CI=1.04-10.2, p=0.05), even after adjusting for confounding factors.^(17)^

Regarding pregnancy morbidity, the PROMISSE study (Predictors of Pregnancy Outcome: Biomarkers in Antiphospholipid Antibody Syndrome and Systemic Lupus Erythematosus), an observational study of pregnancy outcomes in patients with PAPS, SAPS, and healthy individuals, found that LA was a marker of poor gestational outcome, regardless of its association with other aPL antibodies.^(18)^ The authors also described similar outcomes for TP and LA patients with preeclampsia (OR=2.43, 95%CI=1.17-5.04, p=0.02; and OR=2.10, 95%CI=1.14-3.85, p=0.02, respectively) and fetal growth restriction (OR=2.47, 95%CI=1.05-5.80, p=0.04; and OR=1.78, 95%CI=1.16-2.74, p<0.01, respectively).^(18)^ Additionally, in a retrospective study of 79 women with APS, 10 of the 12 unsuccessful pregnancies were TP; however, comparisons did not reach statistical significance when compared with isolated positivity (p=0.08, all single aPL profiles grouped together).^(19)^ These data may explain the similarity of obstetric events between the two groups included in our study.

Finally, hydroxychloroquine (HCQ) is used in APS as an adjunct to anticoagulation, mainly in refractory cases.^(20–23)^ Since this was an observational real-world study and considering that patients from both groups did not have distinct clinical presentations, the higher rates of HCQ found in the TP Group may be explained by the fact that clinicians most likely considered TP a poor prognostic factor, which led to more frequent prescriptions of this medication. However, we acknowledge that the discrepancy in HCQ use between the groups could have confounded the results, potentially masking a true difference in disease severity by mitigating adverse outcomes in the TP Group.

Our study has some limitations. First, it was cross-sectional; therefore, we were unable to establish causality between risk factors and outcomes. Second, the number of patients was relatively low. The reduced sample size may have limited the power of the study to detect true differences between groups, which could partially account for our findings. It may also restrict the generalizability of our results. However, we must consider the rarity of the disease and the strict inclusion criteria. Third, our patients were from tertiary referral hospitals, which may have introduced selection and survival biases. These may have influenced the high frequency of complications and thrombotic outcomes observed herein. Fourth, the TP profile is less prevalent, which impacts the number of patients in this group compared with LA isolated positivity.^(7)^ Fifth, aPL tests were conducted in more than one laboratory; however, the overall distribution of aPL profiles was similar between them. Standardized tests used for diagnosis have comparable results in terms of positivity or negativity, although antibody titers may be more difficult to compare. Sixth, ancestry was based on self-declaration of skin color without further genetic assessment, which may limit generalizability. Finally, LA should preferably be tested without anticoagulants, which was not possible in all patients. Although we aimed for testing to be performed without anticoagulants, the high-risk profile and risk of recurrent thrombosis in many patients made this challenging. Although the results should be interpreted with caution due to the possibility of false-positive results, we performed LA determination in two experienced laboratories and confirmed persistence of this aPL. From another perspective, this study has the strength of including a highly diverse and admixed population. Although we divided the patients in White and non-White groups based on self-declaration, our population includes multiple ancestral backgrounds, including indigenous, African, European, and Asian ancestry. This admixture differs markedly from other published studies and may provide additional insight into APS.

We could not observe significant clinical differences between patients with isolated LA positivity and those with TP.

## CONCLUSION

Although current literature indicates a worse clinical course in antiphospholipid antibodies triple-positive patients, this study did not find a more severe phenotype in this antiphospholipid antibodies profile compared with isolated lupus anticoagulant positivity. Despite these findings, both antibody profiles continue to be associated with a high risk of clinical events and thus require close monitoring. Further studies should focus on different demographic populations to confirm these findings.

## Data Availability

The underlying content is contained within the manuscript.

## References

[1] Barbhaiya M, Zuily S, Naden R, Hendry A, Manneville F, Amigo MC, Amoura Z, Andrade D, Andreoli L, Artim-Esen B, Atsumi T, Avcin T, Belmont HM, Bertolaccini ML, Branch DW, Carvalheiras G, Casini A, Cervera R, Cohen H, Costedoat-Chalumeau N, Crowther M, de Jesus G, Delluc A, Desai S, De Sancho M, Devreese KM, Diz-Kucukkaya R, Duarte-Garcia A, Frances C, Garcia D, Gris JC, Jordan N, Leaf RK, Kello N, Knight JS, Laskin C, Lee AI, Legault K, Levine SR, Levy RA, Limper M, Lockshin MD, Mayer-Pickel K, Musial J, Meroni PL, Orsolini G, Ortel TL, Pengo V, Petri M, Pons-Estel G, Gomez-Puerta JA, Raimboug Q, Roubey R, Sanna G, Seshan SV, Sciascia S, Tektonidou MG, Tincani A, Wahl D, Willis R, Yelnik C, Zuily C, Guillemin F, Costenbader K, Erkan D, ACR/EULAR APS Classification Criteria Collaborators (2023). The 2023 ACR/EULAR Antiphospholipid Syndrome Classification Criteria. Arthritis Rheumatol.

[2] Miyakis S, Lockshin MD, Atsumi T, Branch DW, Brey RL, Cervera R (2006). International consensus statement on an update of the classification criteria for definite antiphospholipid syndrome (APS). J Thromb Haemost.

[3] Pengo V, Ruffatti A, Legnani C, Testa S, Fierro T, Marongiu F (2011). Incidence of a first thromboembolic event in asymptomatic carriers of high-risk antiphospholipid antibody profile: a multicenter prospective study. Blood.

[4] Pengo V, Ruffatti A, Legnani C, Gresele P, Barcellona D, Erba N (2010). Clinical course of high-risk patients diagnosed with antiphospholipid syndrome. J Thromb Haemost.

[5] Sciascia S, Sanna G, Murru V, Roccatello D, Khamashta MA, Bertolaccini ML (2013). GAPSS: the Global Anti-Phospholipid Syndrome Score. Rheumatology (Oxford).

[6] Radin M, Sciascia S, Erkan D, Pengo V, Tektonidou MG, Ugarte A, Meroni P, Ji L, Belmont HM, Cohen H, Ramires de Jesús G, Branch DW, Fortin PR, Andreoli L, Petri M, Rodriguez E, Rodriguez-Pinto I, Knight JS, Atsumi T, Willis R, Gonzalez E, Lopez-Pedrera R, Rossi Gandara AP, Borges Gualhardo Vendramini M, Banzato A, Sevim E, Barbhaiya M, Efthymiou M, Mackie I, Bertolaccini ML, Andrade D, APS ACTION (2019). The adjusted global antiphospholipid syndrome score (aGAPSS) and the risk of recurrent thrombosis: Results from the APS ACTION cohort. Semin Arthritis Rheum.

[7] Laurent C, Ricard L, Nguyen Y, Boffa JJ, Rondeau E, Gerotziafas G (2023). Triple positive profile in antiphospholipid syndrome: prognosis, relapse and management from a retrospective multicentre study. RMD Open.

[8] Galli M, Luciani D, Bertolini G, Barbui T (2003). Lupus anticoagulants are stronger risk factors for thrombosis than anticardiolipin antibodies in the antiphospholipid syndrome: a systematic review of the literature. Blood.

[9] Urbanus RT, de Groot PG (2011). Antiphospholipid antibodies – we are not quite there yet. Blood Rev.

[10] Gebhart J, Posch F, Koder S, Perkmann T, Quehenberger P, Zoghlami C (2015). Increased mortality in patients with the lupus anticoagulant: the Vienna Lupus Anticoagulant and Thrombosis Study (LATS). Blood.

[11] Devreese KM, Ortel TL, Pengo V, de Laat B, Subcommittee on Lupus Anticoagulant/Antiphospholipid Antibodies (2018). Laboratory criteria for antiphospholipid syndrome: communication from the SSC of the ISTH. J Thromb Haemost.

[12] Grundy SM, Stone NJ, Bailey AL, Beam C, Birtcher KK, Blumenthal RS (2019). 2018 AHA/ACC/AACVPR/AAPA/ABC/ACPM/ADA/AGS/APhA/ASPC/NLA/PCNA Guideline on the Management of Blood Cholesterol: Executive Summary: A Report of the American College of Cardiology/American Heart Association Task Force on Clinical Practice Guidelines. J Am Coll Cardiol.

[13] American Diabetes Association Professional Practice Committee (2022). 2. Classification and Diagnosis of Diabetes: Standards of Medical Care in Diabetes-2022. Diabetes Care.

[14] Whelton PK, Carey RM, Aronow WS, Casey DE, Collins KJ, Dennison Himmelfarb C (2018). 2017 ACC/AHA/AAPA/ABC/ACPM/AGS/APhA/ASH/ASPC/NMA/PCNA Guideline for the Prevention, Detection, Evaluation, and Management of High Blood Pressure in Adults: Executive Summary: A Report of the American College of Cardiology/American Heart Association Task Force on Clinical Practice Guidelines. Hypertension.

[15] Chaturvedi S, McCrae KR (2017). Diagnosis and management of the antiphospholipid syndrome. Blood Rev.

[16] Urbanus RT, Siegerink B, Roest M, Rosendaal FR, de Groot PG, Algra A (2009). Antiphospholipid antibodies and risk of myocardial infarction and ischaemic stroke in young women in the RATIO study: a case-control study. Lancet Neurol.

[17] Hernández-Molina G, Espericueta-Arriola G, Cabral AR (2013). The role of lupus anticoagulant and triple marker positivity as risk factors for rethrombosis in patients with primary antiphospholipid syndrome. Clin Exp Rheumatol.

[18] Buyon JP, Kim MY, Guerra MM, Laskin CA, Petri M, Lockshin MD (2015). Predictors of pregnancy outcomes in patients with lupus: a cohort study. Ann Intern Med.

[19] Ruffatti A, Tonello M, Cavazzana A, Bagatella P, Pengo V (2009). Laboratory classification categories and pregnancy outcome in patients with primary antiphospholipid syndrome prescribed antithrombotic therapy. Thromb Res.

[20] Kravvariti E, Koutsogianni A, Samoli E, Sfikakis PP, Tektonidou MG (2020). The effect of hydroxychloroquine on thrombosis prevention and antiphospholipid antibody levels in primary antiphospholipid syndrome: A pilot open label randomized prospective study. Autoimmun Rev.

[21] Schmidt-Tanguy A, Voswinkel J, Henrion D, Subra JF, Loufrani L, Rohmer V (2013). Antithrombotic effects of hydroxychloroquine in primary antiphospholipid syndrome patients. J Thromb Haemost.

[22] Tektonidou MG, Andreoli L, Limper M, Amoura Z, Cervera R, Costedoat-Chalumeau N (2019). EULAR recommendations for the management of antiphospholipid syndrome in adults. Ann Rheum Dis.

[23] Belizna C, Pregnolato F, Abad S, Alijotas-Reig J, Amital H, Amoura Z (2018). HIBISCUS: hydroxychloroquine for the secondary prevention of thrombotic and obstetrical events in primary antiphospholipid syndrome. Autoimmun Rev.

